# The Role of Peroxisome Proliferator-Activated Receptor Gamma and Atherosclerosis: Post-translational Modification and Selective Modulators

**DOI:** 10.3389/fphys.2022.826811

**Published:** 2022-03-02

**Authors:** Liqin Yin, Lihui Wang, Zunhan Shi, Xiaohui Ji, Longhua Liu

**Affiliations:** ^1^School of Kinesiology, Shanghai University of Sport, Shanghai, China; ^2^Department of Medical Imaging, Shanghai East Hospital (East Hospital Affiliated to Tongji University), Tongji University, Shanghai, China

**Keywords:** PPARγ, atherosclerosis, post-translational modifications, selective modulators, cardiovascular disease

## Abstract

Atherosclerosis is the hallmark of cardiovascular disease (CVD) which is a leading cause of death in type 2 diabetes patients, and glycemic control is not beneficial in reducing the potential risk of CVD. Clinically, it was shown that Thiazolidinediones (TZDs), a class of peroxisome proliferator-activated receptor gamma (PPARγ) agonists, are insulin sensitizers with reducing risk of CVD, while the potential adverse effects, such as weight gain, fluid retention, bone loss, and cardiovascular risk, restricts its use in diabetic treatment. PPARγ, a ligand-activated nuclear receptor, has shown to play a crucial role in anti-atherosclerosis by promoting cholesterol efflux, repressing monocytes infiltrating into the vascular intima under endothelial layer, their transformation into macrophages, and inhibiting vascular smooth muscle cells proliferation as well as migration. The selective activation of subsets of PPARγ targets, such as through PPARγ post-translational modification, is thought to improve the safety profile of PPARγ agonists. Here, this review focuses on the significance of PPARγ activity regulation (selective activation and post-translational modification) in the occurrence, development and treatment of atherosclerosis, and further clarifies the value of PPARγ as a safe therapeutic target for anti-atherosclerosis especially in diabetic treatment.

## Introduction

Atherosclerosis is the hallmark of CVD which is a leading cause of death in type 2 diabetes patients, and glycemic control is not beneficial in reducing the potential risk of CVD (Libby et al., 2016; Vallee et al., 2019; Machado-Oliveira et al., 2020). Atherosclerosis is a disease caused by the combination of high oxidative stress, inflammation (Finn et al., 2012), immune response, lipid deposition, and genetic traits (Falk, 2006; Yu et al., 2013; Johnson, 2017). Atherosclerosis is initiated by a large number of abnormally metabolized lipids including apolipoprotein B-containing lipoproteins (apoB LPs) continuously enter into the vascular intima to trigger an inflammatory response dominated by macrophages in the vascular wall (Chistiakov et al., 2015; Tabas, 2017), promote the migration and proliferation of vascular smooth muscle cells (VSMCs) (Durham et al., 2018), cause the vascular wall thickening and the lumen narrowing, and finally develop atherosclerosis (Wang et al., 2015; Bennett et al., 2016).

PPARγ is a ligand-activated nuclear receptor, that regulates glucose and lipid metabolism, endothelial function, and inflammation (Lehrke and Lazar, 2005; Janani and Ranjitha Kumari, 2015). Due to the different selected promoters and alternative shear modes, the PPARγ gene can transcriptionally generate two PPARγ transcript variants, and translate into two isoforms, PPARγ1 and PPARγ2, with PPARγ2 has 30 more amino acid residues at the N-end (Fajas et al., 1997). PPARγ1 is expressed nearly in all cells, while PPARγ2 is mainly expressed in adipocytes and vascular endothelial cells. Nevertheless, PPARγ2 is a more potent transcription activator (Lehrke and Lazar, 2005; Janani and Ranjitha Kumari, 2015). PPARγ plays a crucial role in anti-atherosclerosis by promoting cholesterol efflux (Ozasa et al., 2011; Tsuboi et al., 2020), inhibiting monocytes infiltrating into the vascular intima under endothelial layer (Namgaladze et al., 2013), and inhibiting their transformation into macrophages (Zhang and Chawla, 2004; Charo, 2007; Oppi et al., 2020), inhibiting VSMCs proliferation and migration (Zhang et al., 2011; Durham et al., 2018). PPARγ has emerged as one of the most promising therapeutic targets for cardiovascular complications, and its synthetic ligands (Lim et al., 2015), such as Thiazolidinediones (TZDs) have also been shown to have anti-atherosclerosis function (Viles-Gonzalez et al., 2004; Nakaya et al., 2009). Although their advantages are recognized, the profiles of numerous adverse effects hinder the continued use of these drugs.

To develop a safer and better treatment of cardiovascular complications targeting PPARγ, novel strategies that preserve the “good” potent insulin sensitization, while reducing or eliminating “bad”-related side effects should be used. These novel strategies may include downstream effectors of PPARγ-mediated insulin sensitization, targeting specific post-translational modification (PTMs) (Wang and Tafuri, 2003; Brunmeir and Xu, 2018) of PPARγ, and selective PPARγ modulators (SPPARMs) (Higgins and Depaoli, 2010; Dunn et al., 2011). PTMs such as phosphorylation (Yin et al., 2006; Choi et al., 2011; Montanari et al., 2020), acetylation (Qiang et al., 2012; Kraakman et al., 2018; Liu L. et al., 2020), ubiquitination (Garin-Shkolnik et al., 2014), and sumoylation (Pascual et al., 2005) are all involved in regulating PPARγ activity. These PTMs of PPARγ could regulate its transcription of downstream genes via changing protein conformation, regulating protein interactions, or altering the affinity between receptors and ligands (van Beekum et al., 2009; Brunmeir and Xu, 2018). PPARγ activation requires ligand recognition and binding to receptor regulatory receptor mediated gene transcription (Lehrke and Lazar, 2005; Janani and Ranjitha Kumari, 2015). The binding site and binding region of the ligand with PPARγ determine the conformation of PPARγ and the subsequent changes in cofactor recruitment (Higgins and Depaoli, 2010; Montanari et al., 2020). The selective PPARγ modulators (SPPARMs) is thought to improve the safety profile of PPARγ agonists avoiding TZDs’ adverse reactions.

In this review, we focus on the significance of PPARγ activity regulation (PTMs and SPPARMs) in the occurrence, development and treatment of atherosclerotic diseases, and further clarifies the value of PPARγ as a therapeutic target for anti-atherosclerosis.

## Peroxisome Proliferator-Activated Receptor Gamma and Atherosclerosis

PPARγ has a typical structure of nuclear hormone receptors, including the N-terminal A/B domain, DNA binding domain (DBD) and ligand binding domain (LBD) (Lehrke and Lazar, 2005; Chandra et al., 2008; Janani and Ranjitha Kumari, 2015). Both forms of PPARγ1 and PPARγ2 have a similar structural, except for PPARγ2 containing an N-terminal extension of 28 amino acids (Chandra et al., 2008). PPARγ usually form a heterodimer with Retinoid X Receptor α (RXRαChandra et al., 2008) and binds to PPRE. When the ligand is not bound, PPARγ/RXRα mainly binds to some co-repressors, such as Nuclear Receptor Corepressor (NCoR) or Silencing Mediator of Retinoic Acid and Thyroid Hormone Receptor (SMRT). When the ligand binds to LBD, it will change the conformation of PPARγ, and the co-repressors will be replaced by some co-activators, e.g., cAMP responsive element binding protein (CREBP), PPARγ coactivator-1 (PGC-1), Steroid Receptor Coactivator (SRC), and CBP/P300 (Lehrke and Lazar, 2005; Chandra et al., 2008). The recruited cofactors vary in their transcriptional regulatory target genes, and their transcriptional levels and biological functions will change accordingly.

**FIGURE 1:**
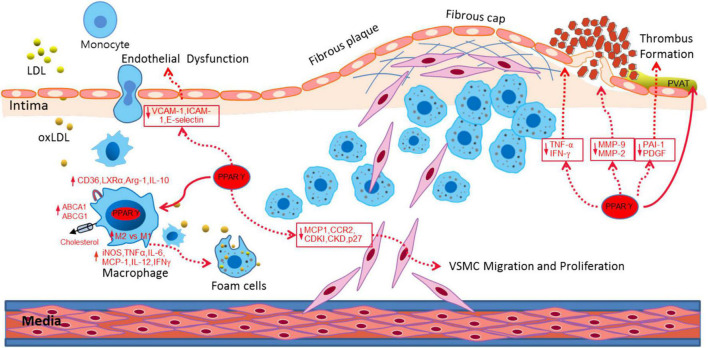
PPARγ attenuated atherosclerosis through different aspects, including alleviating endothelial dysfunction, promoting cholesterol efflux, inducing M1-M2 transition, inhibiting VSMC migration and proliferation and stabilizing the fibrous cap and plaque.

PPARγ not only participates in fat formation, lipid and glucose metabolism, but also plays an important role on vascular biology and inflammation, and the development of atherosclerosis (Kvandová et al., 2016; Hernandez-Quiles et al., 2021). PPARγ has anti-atherosclerotic effects through the following aspects (Figure 1): (1) PPARγ regulates the expression of cell adhesion molecules, such as inducible nitric oxide synthase (Chen et al., 2001), intracellular cell adhesion molecule-1(ICAM-1), vascular cell adhesion molecule-1(VCAM-1) (Babaev et al., 2005), and inhibit endothelial cells activation and attenuation of monocyte chemoattractant protein 1 (MCP-1), matrix metalloproteinase 9 (MMP9), and metallopeptidase inhibitor 1 (TIMP-1) which induced monocyte migration across endothelial cells (Verrier et al., 2004; Calabrò et al., 2005); (2) PPARγ activates the PPARγ/liver X receptor α (LXR-α) pathway to stimulate the expression of cholesterol efflux-related genes– ATP binding cassette transporter A1 (ABCA1) (Akiyama et al., 2002) and Acy1 Coenzyme A: Cholesterol Acyltransferases (ABCG1) (Sueyoshi et al., 2010; Srivastava, 2011), accelerates the efflux of cholesterol from macrophages (Liu D. et al., 2020; Oppi et al., 2020), therefore inhibits the formation of foam cells (Li et al., 2004; Zhang et al., 2021); (3) PPARγ inhibits the expression of pro-inflammatory factors, such as TNF-α (Zhang et al., 2014), IL-6, IL-18 (Chen et al., 2008), and induces the macrophages differentiation into an anti-inflammatory M2 phenotype (Oppi et al., 2020). And the expression of M2 markers, such as MR, AMAC1, and IL-10 levels correlate positively with the expression of PPARγ (Bouhlel et al., 2007). Therefore, PPARγ can improve the inflammatory response of cardiovascular cells, inhibit plaque formation, and maintain plaques stability. (4) PPARγ inhibits VSMCs proliferation and migration by suppressing TLR4-mediated inflammation (Gu et al., 2019) and ultimately attenuates intimal hyperplasia after carotid injury (Meredith et al., 2009; Osman and Segar, 2016). Meanwhile, PPARγ prevents the degradation of cyclin-dependent kinase inhibitors (CDKIs) and p27 induced by growth factors, and inhibits the formation of cyclin-dependent kinase complex (Cyclin-CDK), thereby inhibits proliferation, migration, and apoptosis (Law et al., 2000; Fu et al., 2001). (5) PPARγ inhibits the expression of MMP-9 and MMP-2 that can decompose collagen and fibers in macrophages (Luo et al., 2007; Reinhold et al., 2020), reduces the fragility of the fiber cap and enhances the stability of the plaque. Therefore, the functional regulation of PPARγ is of very important for the prevention and treatment of atherosclerosis.

## Post-Translational Modifications of Peroxisome Proliferator-Activated Receptor Gamma and Atherosclerosis

The PPARγ transcriptional activity regulates in diverse ways, including protein expression levels, ligands, and transcriptional cofactors. PTMs of proteins can alter protein conformation, regulate protein interactions, and alter the affinity between receptors and ligands, thus regulating the transcription of downstream genes (Brunmeir and Xu, 2018).

### Phosphorylation

**FIGURE 2:**
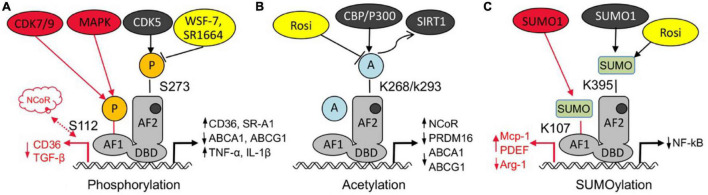
Post-translational modification of PPARγ regulates atherosclerosis. **(A)** Both phosphorylation of PPARγ at S112 by CDK7/9 or MAPK and phosphorylation of PPARγ at S273 accelerates foam cell formation and atherosclerosis through different signaling pathway, some PPARγ agonist (WSF-7, SR1664) can block cdk5 mediated Ser273 phosphorylation. **(B)** Acetylation of PPARγ at K268/K293 increases atherosclerosis through upregulating ABCA1, ABCG1, and NcoR but inhibiting PRDM16,while deacetylation of PPARγ at K268 and K293 alleviates atherosclerosis, while PPARγ agonist rosiglitazone (Rosi) could deacetylate PPARγ at K268/K293. **(C)** Sumoylation of PPARγ at K107 promotes VSMCs proliferation and migration, but sumoylation of PPARγ at K395, such as by Rosi, has anti-inflammation effect.

The phosphorylation regulation of PPARγ is one of the main ways to regulate its activity (Montanari et al., 2020). With different stimuli, PPARγ could be phosphorylated at different sites and resulting diverse biological effects (Choi et al., 2011). Cyclin-dependent kinase (CDK) (Choi et al., 2010, 2011; Laghezza et al., 2018) and mitogen-activated protein kinase (MAPK) (Yin et al., 2006; Yang et al., 2013; Ge et al., 2018) are involving in the phosphorylation of PPARγ, and the main sites include Ser273 (245 in isoform 1) (Dias et al., 2020; Hall et al., 2020) and Ser112 (Ser82 in isoform 1) (Figure 2A; Ge et al., 2018). CDK5-mediated phosphorylation of PPARγ S273 results in a decrease in its transcriptional activity and adipogenesis. One of TZDs’ major side effects is due to its activation of PPARγ in adipose tissue, and high-fat diet increased CDK5-mediated of PPARγ phosphorylation, which was negatively associated with TZDs’ insulin sensitization in humans. Meanwhile PPARγ phosphorylation can up-regulate lipid uptake of CD36 and SR-A1 related proteins, inhibit cholesterol efflux ABCA1 and ABCG1 related proteins, induce the expression of TNF-α, IL-1β and other inflammatory factors, and promote the formation of foam cells to accelerate the process of atherosclerosis (Choi et al., 2010, 2011; Banks et al., 2015). PPARγ phosphorylation by CDK5 may contribute to its dissociation with PGC1α and TIF2 coactivators but interaction with SMRT and NCoR corepressors (Dias et al., 2020). NCoR can regulate the phosphorylation of PPARγ on Ser 273 by stabilizing CDK5 (Li et al., 2011). Mice with fat cell NCoR knockout (NCoR^–/–^) improves glucose tolerance and insulin sensitivity and reduces macrophage infiltration and inflammation (Ribeiro Filho et al., 2019). As from CDK5 studies, compounds can be designed to alter specific PTMs of PPARγ to partly prevent disturbed fat metabolism while retaining anti-diabetic potency. CDK9/CDK7-mediated phosphorylation of S112 can increase the transcriptional activity of PPARγ and promote adipocyte differentiation (Iankova et al., 2006; Li et al., 2017; Ge et al., 2018).

MAPK can phosphorylate PPARγ in the AF1 region (PPARγ2 Ser112, PPARγ1 Ser82), inhibiting the ligand binding and changing the recruitment of co-factors, and then change the transcriptional activity (Yin et al., 2006; Yang et al., 2013; Ge et al., 2018). MAPK-mediated phosphorylation of PPARγ, which promotes the formation of foam cells by macrophages exposed to ox-LDL (Yin et al., 2006). Growth factors can phosphorylate PPARγ by the MAPK signaling pathway and reduce the transcriptional activity of PPARγ (Yin et al., 2006), such as epidermal growth factor (EGF) and platelet-derived growth factor (PDGF) (Osman and Segar, 2016). In addition, Choi et al. (2015) recently described the phosphorylation of Y78, is also important for the cytokine and chemokine gene expression’s regulation. PPARγ phosphorylation can alter its transcriptional activity, and the blockage of PPARγ phosphorylation is related to improve insulin sensitization (Choi et al., 2014). However, PPARγ phosphorylation mediated by different enzymes, conformational changes at different sites can cause the recruitment response of different cofactors, its role in atherosclerosis and its mechanism need to be further studied, and also provide ideas for the drug design of PPAR ligand.

### Acetylation

Acetylation of PPARγ is a ligand- independent activation of PPARγ. Qiang et al. (2012) showed that five lysine residues (K98, K107, K218, K268, and K293) could be acetylated, of which two K268/K293 could be deacetylated by TZD rosiglitazone (Rosi) via activation of the NAD (Nicotinamide adenine dinucleotide)-dependent deacetylase sirtuin-1 (SIRT1) deacetylase (Figure 2B; Kraakman et al., 2018). PPARγ is acetylated by p300 or CBP (Kim et al., 2006), and it may play an important role in lipid synthesis (Tian et al., 2014). Acetylation of PPARγ at K268/K293 increases atherosclerosis through upregulating ABCA1, ABCG1, and NcoR but inhibiting PRDM16, while deacetylation of PPARγ at K268 and K293 alleviates atherosclerosis.

PPARγ deacetylation on K268 and K293 induces brown remodeling of white adipose tissue and reduces the adverse effects of TZDs while maintaining insulin sensitization (Qiang et al., 2012; Liu L. et al., 2020). Deacetylation of PPARγ can selective regulation the target genes, it could inhibit aP2, Cd36, upregulation genes ucp1 and adipsin on lipid oxidative genes cpt1a (Kraakman et al., 2018). PPARγ deacetylation improves endothelial function with diabetes treatment. The aortic arch lesion size was reduced in 2KR (K268 and K293) LDLr^–/–^ mice, the expression of iNOS, Nox2, and IL-6 in endothelial cells were decreased, while the side effects of TZD, including fluid retention and bone loss were reduced (Liu L. et al., 2020). Deacetylation of PPARγ inhibits the cholesterol efflux PPARγ/LXRα/ABCA1 pathway (Cao et al., 2014; Yang et al., 2015), increased production of proinflammatory M1 macrophages and promotes the development of inflammatory response (Chen et al., 2008, 2010), leading to the onset and development of atherosclerosis. From a mechanistic perspective, deacetylated PPARγ preferentially interacts with PRDM16 and disrupts the binding of the transcriptional corepressor NCoR (Qiang et al., 2012). Therefore, manipulating PPARγ acetylation is a promising therapeutic strategy to anti-atherosclerosis.

### Sumoylation

PPARγ sumoylation with SUMO1 modification of K107 (K77 in PPARγ1) (Figure 2C), and ubiquitin carrier protein 9 (Ubc9) and PIAS1 (protein inhibitor of activated STAT1) are involved as PPARγ specific E2 binding enzymes and E3 ligases, respectively (Lim et al., 2009). Sumoylation of PPARγ K107 inhibits its transcriptional activity, and is enhanced by the K107 mutation (K107R). PPARγ sumoylation at K107 position strongly inhibited VSMCs proliferation and migration (Katafuchi et al., 2018), and reduced neointimal formation after balloon injury (Lim et al., 2009). Desumoylation at K107 in PPARγ may inhibit serum-stimulated VSMCs proliferation (Lim et al., 2009; Osman and Segar, 2016), might play an important role against atherosclerosis. Moreover, K107R improves insulin sensitivity without body weight gain or adiposity (Wadosky and Willis, 2012; Pourcet et al., 2013). However, some studies have shown that K107 sumoylation plays an important role in the anti-inflammatory response triggered by apoptotic cells, possibly by stabilizing the co-inhibitor NCoR on the target gene (Jennewein et al., 2008; Lu et al., 2013). The sumoylation modification of PPARγ1 inhibits the M2 polarization of macrophages by inhibiting the transcription of Arg-1 (Haschemi et al., 2011). Pascual et al. (2005) shows that the PPAR agonist TZD can exert anti-diabetic and anti-atherosclerotic effects through the NF-kB inflammatory pathway, the first response is associated with TZD-mediated SUMO1 modification of K365 (K395 in PPARγ2) followed by targeted regulation of the PPAR co-cofactor NCoR. The exact biological role of the two modifications in anti-inflammatory responses, especially the potential functional overlap, remains to be determined. Nevertheless, targeting PPARγ sumoylation may provide a novel mechanism for anti-atherosclerosis.

### Ubiquitination

Ubiquitination modification cannot only regulate the proteasome-mediated degradation of target proteins, but also serve as a “scaffold” to recruit other proteins to form signal complexes. PPARγ undergoes conformational changes after binding to the ligand. On the one hand, Makorin RING finger protein 1 (MKRN1) (Kim et al., 2014) and Seven *in absentia* homolog 2 (SIAH2) (Kilroy et al., 2012) services as PPARγ E3 ligases, targeting PPARγ for proteasomal degradation. Ubiquitination of PPARγ on K184 and K185 inhibits its activity in mature 3T3-L1 adipocytes (Kilroy et al., 2012; Kim et al., 2014). On the other hand, it can also recruit the binding of ubiquitination-related enzymes and induce proteasome-dependent degradation, thereby negatively regulating the transcriptional activity of PPARγ. Rosiglitazone reduces the inflammatory response in diabetic plaques, less ubiquitin, proteasome 20S, TNF-α, and NF-κB, ubiquitin-proteasome activity with diabetic plaque NF-κB-mediated inflammatory response is involved (Marfella et al., 2006). This study strengthens the earlier findings on PPARγ regulation through modulation of its stability. Currently, the use of ubiquitination modifications for the regulation of PPARγ transcriptional activity is still controversial (Watanabe et al., 2015). It has been reported that the ubiquitin-proteasome pathway can mediate the protein renewal of PPARγ (Li et al., 2016), a process that is required for the efficient transcription of its downstream genes, while the ubiquitin activase inhibitor E1 inhibitor, and the proteasome inhibitor MG-132 can cause a decrease in PPARγ transcriptional activity.

## Selective PPARγ Modulators and Atherosclerosis

PPARγ ligands are generally lipid-derived compounds with natural and synthetic properties, and different ligands have different affinity-binding receptors and activate the receptors. Synthetic ligands TZDs reduce atherosclerosis in certain mouse models (Martens et al., 2002). However, severe side effects associated with TZD use, such as weight gain, fluid retention, bone loss, cardiovascular disk, etc., restricts the use of TZDs (Zinn et al., 2008). In order to maximize the PPAR-mediated insulin sensitization and to minimize the occurrence of related adverse reactions, the concept of “selective PPAR regulator (selective PPARγ modulators, SPPARMs)” was proposed and developed (Higgins and Depaoli, 2010; Camejo, 2016). In contrast to rosiglitazone, SPPRAMs has similar or different PPAR receptor binding sites, or has different affinity or specificity of recruitment receptor cofactors, or a range of target genes where PPAR are biased and selective for regulating transcription. The currently found SPPRAMs that associated with atherosclerosis mainly consists of three types: partial PPARγ agonist, dual PPAR α/γ agonist, and non-agonist PPARγ ligand.

### Partial Peroxisome Proliferator-Activated Receptor Gamma Agonist

In fact, partial PPARγ agonist is different from the classic TZDs, with rosiglitazone as the “full agonist.” It is generally believed that 20–60% of rosiglitazone efficacy is a partial activator (Table 1), such as GQ-177 (Silva et al., 2016), S 26948 (Carmona et al., 2007), WSF-7 (Zhang et al., 2020) lobeglitazone (Lim et al., 2015), and INT131 (Xie et al., 2017). LDLr^–/–^ mice treated with GQ-177 can significant decrease the VLDL, LDL fractions and increase mean HDL, Glut4 levels, increased the expression of apoA1, CD36, ABCA1, SR-B1, and ABCG5 in hepatic, contrary to rosiglitazone, GQ-177 did not affect fat accumulation and bone mineral density (Silva et al., 2016). It was shown that TZDs facilitated the transport of BM-derived circulating progenitor cells to adipose tissue and their differentiation into multilocular adipose cells (Crossno et al., 2006). Meanwhile, Hu et al. (2021) found RANKL from bone marrow adipose lineage cells promoted osteoclast formation and bone loss. S26948 improves lipid parameters (LDLs, VLDL) and reduces atherosclerotic lesions in ob/ob male C57BL/6 mice (Carmona et al., 2007). WSF-7 upregulated PPARγ-responsive genes, such as adiponectin and Glut4, inhibits PPARγ phosphorylation at Ser273 by obesity and enhances insulin sensitivity in 3T3-L1 Adipocytes (Zhang et al., 2020). Lobeglitazone inhibits the VSMCs proliferation and migration, reduces the vascular cells adhesion, NF-kB p65 translocation, and improves circulating factors related to atherosclerosis, then reduced neointimal formation significantly in balloon injury rat carotid arteries in ApoE^–/–^ mice (Lim et al., 2015; Song et al., 2021). In the presence of pro-inflammatory stimulation, Lobeglitazone effectively inhibited expression of pro-inflammatory gene expression in macrophages and adipocytes (Sohn et al., 2018). The latest study has found that macrophages targeted PPARγ activator Lobeglitazone could rapidly stabilize a coronary artery-sized inflammatory plaque (Song et al., 2021). Both non-clinical and clinical studies have demonstrated that INT131 have the potential to separate insulin-sensitizing actions and undesirable side effects in Patients With Type 2 Diabetes (DePaoli et al., 2014), and it also has the potential to decrease free fatty acids, increase HDL-C (Dunn et al., 2011). However, there is still no relevant study on the effect of INT131 on atherosclerosis.

**TABLE 1:** Selective PPARγ modulators associated with atherosclerosis.

Class	Compound	Target gene	Function	Mechanisms	References
Partial PPARγ agonist	GQ-177	apoA1, ABCA1, SR-B1 ABCG5, ABCG8, and HDL-c	Inhibits the progression of atherosclerotic lesions. not affect fat accumulation, bone mineral density.	Hydrophobic contacts with residues from arm II.	Silva et al., 2016
	S 26948	LDLs, VLDL, LPL, aP2↓, UCP1	Promotes cholesterol transfer and reduce lesions surface.	No-recruit DRIP205 or PCG-1α.	Carmona et al., 2007
	WSF-7	Adiponectin and Glut4↑	Enhances insulin sensitivity, Reduce the fat accumulation.	Inhibits PPARγ Ser273 phosphorylation.	Zhang et al., 2020
	Lobeglitazone	hsCRP, MCP-1, ABCA1, leptin↓	Inhibits VSMC proliferation, Powerful anti-inflammatory effect.	Additional hydrophobic contacts with the Ω-pocket.	Lim et al., 2015

Dual PPAR α/γ agonist	GQ-11	Mcp-1,VLDL-C↓HDL-C, Apoa1↑, ABCA1, Sr-b1, IL-10↑	Ameliorated insulin sensitivity, promotes cholesterol transfer, no body weight gain.	Hydrogen bond with the PPARγ residue Ser289 at helix 3.	Silva, 2018
	P633H	ACO, aP2,	Not reported with atherosclerosis.	Not reported.	Chen et al., 2009
	C333H	TG, T-CHO, FFA	Reduces blood lipid and glucose concentration	Not reported.	Xu et al., 2006
	LT175	Glut4, Adipoq, Fabp4,NCoR1, CD36↓	Modulating lipid and glucose metabolism, avoiding weight gain	Impaired the recruitment of CBP coactivator	Gilardi et al., 2014
	Compound 3q	VCAM-1, MCP-1, CD36, P-selectin↑	Increases atherogenesis.	Not reported.	Calkin et al., 2007
	Tesaglitazar	SAA, NFκB, ICAM-1, MCP-1↓	Reduce LDL-C, less peripheral edema and body weight gain; heart failure and myocardial ischemia	Acetylation/deactivation of cardiac PGC-1α	Zadelaar et al., 2006

Non-agonist PPARγ	SR1664	Not affect aP2, Glut4, Lpl, CD36	Anti-diabetic, without promoting fluid retention or altering bone formation	Directly block Cdk5 dependent phosphorylation of PPARγ Ser273, do not stabilize helix 12	Choi et al., 2011
	UHC1	TNF-α, LPS, IL-1β, IL-6, MCP-1, IL-10↑	Inhibits the inflammatory responses in adipocytes and macrophages.	Directly block cdk5 mediated PPARγ K395 phosphorylation	Choi et al., 2014
	MSDC		Insulin sensitization, regulate the lipid metabolism	Target Mitochondrial pyruvate carrier2 (MPC2).	Colca et al., 2018

Full agonist-TZDs forms a key hydrogen bond with the side chain of Y473 on helix 12, mainly interacts with residues from arm I in the ligand binding pocket, to enhance the binding affinity of coactivators/weaken corepressors, inducing transcriptional activation (Brust et al., 2018). Unlike full agonist TZDs, some partial agonists have different binding mode, such as GQ-177 interacts through hydrophobic contacts with residues from arm II (Barros et al., 2010), lobeglitazone makes additional hydrophobic contacts with the Ω-pocket. There are also some partial agonists having similar binding sites to TZDs, but the recruiting cofactors were different. For example, INT131 activates PPARγ, but does not recruit the cofactor MED1 (key factor in regulating adipogenesis) (Lee et al., 2012; Xie et al., 2017), S26948 is unable to recruit DRIP205 or PCG-1α (Key genes in regulating gluconeogenesis), so that it selectively reduces blood glucose without the obvious adverse effects of adipogenesis (Carmona et al., 2007; Sohn et al., 2009). Compared to rosiglitazone, Lobeglitazone strongly blocks the phosphorylation of PPARγ at Ser245, but the pharmacological effects of this translational modification change need further studied (Jang et al., 2018). Although the exact mechanism beyond these effects remains to be determined, partial agonist might represent a new class of therapeutic molecules for the treatment of atherosclerosis.

### Dual Peroxisome Proliferator-Activated Receptor α/γ Agonist

Dual PPARα/γ agonist has been focusing on the activation of both PPARα and PPARγ, which may provide a wider range of metabolic benefits. Studies have shown that most of the Dual PPARα/γ agonist play an active role in anti-atherosclerosis, such as GQ-11 (Silva, 2018), P633H (Chen et al., 2009), C333H (Xu et al., 2006), LT175 (Gilardi et al., 2014), and Tesaglitazar (Zadelaar et al., 2006; Chira et al., 2007; Table 1). GQ-11 improved insulin sensitivity and enhanced Glut4 expression in the adipose tissue, meanwhile the levels of MCP-1were reduced and the levels of IL-10 were increased. Furthermore, it also upregulation of Apoa1 and ABCA1 gene expression, then reduced triglycerides and VLDL cholesterol and increased HDL cholesterol in LDLr^–/–^ mice (Silva et al., 2018). P633H can be accompanied by the upregulation of ACO and aP2 expression in db/db and KK-A*^y^* mice, and is targeted to regulate PPARα in the liver and PPARγ in adipose tissue, respectively (Chen et al., 2009). C333H efficiently reduced blood lipid and glucose concentration in db/db mice (Xu et al., 2006). LT175 activates PPARγ in adipocytes, increases the expression of PPARγ target gene Glut4 and Adipoq in 3T3-L1 adipocytes and in a mouse model. Moreover, LT175 can also activate PPARα in the liver, trigger triglyceride and fatty acid catabolism, and achieve to eliminate the side effects of some conventional PPAR agonists (Gilardi et al., 2014). However, such effects are not clear in human situation. Tesaglitazar reduces atherosclerosis, reduces macrophage inflammation, number of adhesion monocytes and nuclear factor activity of the vessel wall (Zadelaar et al., 2006). However, some Dual PPARα/γ agonist such as compound 3q may accelerate atherosclerosis, it may related to the increase expression of the vascular endothelial activation and inflammation markers, such as P-selectin, MCP-1, VCAM-1, and CD36, that are also associated with plaque complexity (Calkin et al., 2007). Dual agonists such as Tesaglitazar also showed better insulin sensitization effects as well as the prevention of atherosclerosis progression in clinical studies, but due to adverse side effects, including heart failure and myocardial ischemia, it has been discontinued in phase III clinical trials (Yamaguchi et al., 2014).

The PPARγ agonists shown to increase adipogenesis and body weight, whereas PPARα agonists counteract these effects by decreasing food intake and fat deposits. However, both its PPAR binding mode and its downstream targeting will change accordingly. LT175 impaired the recruitment of CBP coactivator, Tesaglitazar is accounted for by inhibition of both expression and acetylation/deactivation of cardiac PGC1α both in healthy C57BL/6 and diabetic db/db mice. Consistent with other partial agonists, GQ-11 only hydrogen bond with the PPARγ residue Ser289 at helix 3, which could reflect in weak PPARγ agonistic activities, and also interacts and weakly activates PPARα (Silva, 2018). Most of the dual PPARα/γ agonists, although they can improve insulin resistance as the full agonists, and do not have similar weight gain, negative bone effects, but it will appear adverse effects on the urothelial, renal, and cardiovascular system (Kaul et al., 2019; Chen et al., 2021). For the adverse side effects of PPARγ agonists in fluid retention, most studies have shown that this effect was due to increased reabsorption of sodium and water by the renal tubules, but the role of specific renal unit segments and sodium carriers was unclear. PPARγ-induced EGF receptors and non-genomic trans-activation of downstream extracellular signal-modulating kinases (ERKs) may augment sodium reabsorption in the proximal tubule (Beltowski et al., 2013). TZDs-like compounds significantly inhibited PPARγ phosphorylation in Ser112 while telmisartan did not (Kolli et al., 2014). Thus, telmisartan did not have a significant effect on osteoclast differentiation and osteogenesis. Dual PPARα/γ Tesaglitazar activation inhibits SIRT1-PGC1α axis and causes cardiac dysfunction. It was showed that this cardiac dysfunction was associated with reduced PGC1α expression. These effects are related to competition between PPARα and PPARγ to regulate Ppargc1a gene expression and to reduce cardiac SIRT1 expression (Kalliora et al., 2019). PGC1α is a regulator of mitochondrial function in thermogenic tissues, such as brown fat. Lehman et al. (2000) also found cardiac-specific overexpression of PGC1α in mice lead to uncontrolled mitochondrial proliferation in cardiomyocytes, resulting in loss of sarcomere structure and dilated cardiomyopathy. Inhibiting PGC1α on dual PPARα/γ activation is potentially as a key event that mediates the cardiotoxic effect, which would provide a guide for design of future PPAR agonists.

### Non-agonist Peroxisome Proliferator-Activated Receptor Gamma Ligand

Non-agonist (Antagonists) PPARγ ligand, also known as PPAR modulators, exhibit high affinity but do not activate PPARγ. The Antagonists consists of two main categories, and one is known as PPARγ phosphorylation inhibitors, such as SR1664 (Cariou et al., 2012; Dias et al., 2020) and UHC1 (Choi et al., 2014). Taken SR1664 as an example, it has basically no transcriptional activation effect on PPARγ, but has a high affinity with PPARγ and belongs to a phosphorylation inhibitor, blocking the CDK5-mediated phosphorylation of PPARγ in Ser273 (Laghezza et al., 2018; Dias et al., 2020). SR1664 did not stimulate lipid accumulation or adipogenesis gene expression (such as aP2, Glut4, Lpl, CD36) in differentiating fat cells. UHC1 blocking CKD5-mediated PPARγ phosphorylation at position K395 in LBD, reducing macrophages inflammatory factor LPS-induced nitric oxide (NO) production both *in vitro* and in HFD-fed mice (Choi et al., 2014; Ribeiro Filho et al., 2019). Comparison with conventional full agonists, these antagonists do not stabilize helix 12 and display negligible changes in activation, but make unfavorable interactions with F282 on helix 3 (Asteian et al., 2015). Another class of PPAR modulators is relatively special, with structurally TZDs analogs, but with little effect on PPARγ, represent the compound as MSDC. It have been showed a potential therapeutic avenue for treating non-alcoholic steatohepatitis, improve the insulin resistance effect, regulate the lipid metabolism (Colca et al., 2018). However, the current study of atherosclerosis has not been reported. Its target of action was reported as a line Mitochondrial pyruvate carrier 2 (MPC2), which is the mitochondrial target of thiazolidinediones (mTOT) (Vigueira et al., 2017). Although not directly stimulated to PPARγ, but still has the potency of insulin sensitization.

## Conclusion and Perspective

PPARγ plays a crucial role in anti-atherosclerosis, PPARγ-mediated anti-atherosclerosis depends on the basic expression level and activity level of PPARγ. At the same time, PPARγ transcription activity is no longer the only criterion, and the mode of action of the compound and PPARγ is the key to PPARγ activity. Both protein post-translational modification and selective modulators are different modes of PPARγ activity regulation. The mode of action between the compound and PPARγ determines the intensity and breadth of PPARγ-mediated transcriptional activation.

However, there are still many questions to be solved, such as the proteases involved in various post-translational modifications, and the protein interactions and downstream target genes regulated by these modifications are still unclear. In addition to the identified post-translational modifications, novel modification patterns or modification sites are still to be discovered. Different post-translational modifications may also interact and form closely related network, which provides a strong guarantee for the fine control of protein functions. The post-translational modifications of proteins are usually reversible, while the de-modification of PPARγ is relatively lagging behind. Transcriptional alteration of post-translational modifications is an innovative idea of new agonist, and only phosphorylation is partially applied to PPARγ agonist, deacetylation and sumoylation has not been involved. How to selectively activate partial downstream targets of PPARγ to protect from atherosclerosis and relative metabolic diseases as well as reducing adverse effects deserve further investigation.

## Author Contributions

LY, LW, ZS, XJ, and LL contributed to the literature search. LY, LW, ZS, and LL wrote and revised the manuscript. LL designed the frame of this manuscript. All authors contributed to this manuscript and approved the submitted version.

## Conflict of Interest

The authors declare that the research was conducted in the absence of any commercial or financial relationships that could be construed as a potential conflict of interest.

## Publisher’s Note

All claims expressed in this article are solely those of the authors and do not necessarily represent those of their affiliated organizations, or those of the publisher, the editors and the reviewers. Any product that may be evaluated in this article, or claim that may be made by its manufacturer, is not guaranteed or endorsed by the publisher.
